# IFN-γ-Mediated Innate Immunity in Females Is Less Efficient than That in Males in Restricting Cerebral Proliferation of *Toxoplasma* Tachyzoites During Reactivation of Chronic Infection with the Parasite

**DOI:** 10.3390/cells15201877

**Published:** 2026-10-15

**Authors:** Rajesh Mani, Eri Ochiai, Qila Sa, Yasuhiro Suzuki

**Affiliations:** Department of Microbiology, Immunology and Molecular Genetics, University of Kentucky College of Medicine, Lexington, KY 40536, USA; rajesh.mani@uky.edu (R.M.); nql58241@nifty.com (E.O.); saqila25@gmail.com (Q.S.)

**Keywords:** innate immunity, gender, male, female, IFN-γ, *Toxoplasma*, cerebral infection

## Abstract

Female AIDS patients infected with *Toxoplasma gondii* have increased risks of developing toxoplasmic encephalitis caused by reactivation of chronic infection with this parasite when compared to males. In the present study, we compared the capability of innate immunity to limit proliferation of tachyzoites (the acute stage form) of *T. gondii* in the brain during reactivation of the infection between genders using SCID mice deficient in T and B cells. SCID mice were infected and treated with sulfadiazine to control tachyzoite proliferation during the acute stage of infection and establish chronic infection in their brains, and thereafter, sulfadiazine was discontinued to initiate reactivation of the infection. Notably, markedly higher levels of mRNA for tachyzoite-specific SAG1 and LDH1 as well as greater numbers of the areas associated with tachyzoite proliferation were detected in the brains of females than in the brains of male SCID mice during reactivation of the infection. Whereas IFN-γ-mediated protective immunity is required for preventing reactivation of cerebral *T. gondii* infection, expression levels of mRNA for IFN-γ, the IFN-γ receptor, the key mediator of the IFN-γ-mediated signaling pathway (STAT1), and effector molecules of IFN-γ-mediated protective immunity did not differ between the brains of male and female SCID mice. Cerebral mRNA levels for IL-1β and TNF-α also did not differ between the sexes of those mice even when tachyzoite proliferation became accelerated in the brain of the females. Notably, cerebral mRNA levels for immunosuppressive arginase 1 (Arg1) became significantly greater in female than in male SCID mice during reactivation of the infection, and increased Arg1 mRNA levels significantly correlated with increased levels of tachyzoite-specific SAG1 and LDH1 mRNA levels in the brains of only females of these mice. These results indicate that IFN-γ-mediated innate immunity to prevent cerebral tachyzoite proliferation is less efficient in female than in male SCID mice during reactivation of *T. gondii* infection, and that increased expression of Arg1 most likely contributes to the susceptibility of female SCID mice to reactivation of the infection.

## 1. Introduction

*Toxoplasma gondii* is an obligate intracellular protozoan parasite that can infect a variety of mammals including humans [1]. During the acute stage of infection, tachyzoites (the acute stage form) proliferate within various types of nucleated cells in the hosts, and this infection can cause various diseases including lymphadenopathy and congenital infection in the fetuses [2]. IFN-γ-mediated protective immunity is essential for controlling the proliferation of tachyzoites [3,4]. However, IFN-γ-mediated protective immunity cannot completely eradicate the parasite during the acute stage of infection, and portions of tachyzoites transform into cysts containing bradyzoites (the chronic stage form) for establishing a chronic infection [2]. The brain is the organ where *T. gondii* preferentially establishes the chronic infection, although the cysts can be formed in other organs [2]. Within the cysts, bradyzoites slowly proliferate during the chronic stage of infection. This persistent infection is abundant in humans worldwide, including in developed countries, with one-third of the human population estimated to be infected with this parasite [2,5,6]. Importantly, when the immune system of the individuals chronically infected with *T. gondii* becomes suppressed by HIV infection, neoplasms, or immunosuppressive treatments for organ transplantations, this infection can reactivate and cause potentially life-threatening toxoplasmic encephalitis (TE) [2]. TE is the most common cerebral opportunistic infectious disease in AIDS patients [2,7,8,9], and this disease is a serious health problem in AIDS patients in both developing [10,11,12] and developed countries [13,14,15].

Notably, a large-scale epidemiological study conducted by the AIDS in Europe Study Group originally reported that the incidence of TE is significantly higher in female than male AIDS patients [16]. Consistently, a more recent study performed in Italy also demonstrated that female sex is associated with a significantly increased risk of developing TE in AIDS patients [17]. However, the mechanisms underlying the increased susceptibility of females to the development of TE remain unknown. Elucidating the mechanisms that increase the susceptibility of females to TE could provide a valuable pathway to improve the prevention and treatment of this serious disease in AIDS patients and possibly other immunocompromised individuals.

Regarding protective immunity against reactivation of chronic cerebral infection with *T. gondii*, IFN-γ is required for preventing this disease [18,19]. Importantly, our recent study revealed that IFN-γ production by brain-resident (irradiation-resistant [non-hematopoietic]) cells is critical for promptly activating both innate and T cell-mediated immunity in the brain to prevent reactivation of the infection [20]. We also identified that microglia and blood-derived macrophages produce IFN-γ in the brain during reactivation of the infection [21]. Based on the increased susceptibility of female immunocompromised AIDS patients to reactivation of cerebral *T. gondii* infection when compared to males, it is possible that innate immune responses in the brain of female individuals with *T. gondii* infection are less effective than those in males in suppressing reactivation of the infection. Thus, in the present study, we examined whether the efficiency of innate immunity to limit reactivation of *T. gondii* infection in the brain differs between females and males using SCID mice deficient in T and B cells. Mice were used in this study because mice are the mammalian animal model in which the largest amount of immunological information is available. We identified that innate immunity in female SCID mice is markedly less capable of restricting cerebral tachyzoite proliferation than that of the males during reactivation of the infection. Our study also revealed that a significant decrease in the efficiency of the IFN-γ-mediated protective innate immunity to inhibit tachyzoite growth occurs in the brains of female SCID mice during the progression of the disease, and that increased cerebral expression levels of arginase 1 (Arg1) correlate with increase tachyzoite proliferation in the brains of only the female SCID mice.

## 2. Materials and Methods

### 2.1. Mice

Males and females of C3H/HeSnJ-background SCID mice (the *H-2^k^* haplotype) were obtained from the Jackson Laboratory (Bar Harbor, ME). Age-matched males and females were used for each experiment, and they were 8–10 weeks old when used in the experiments. There were 4 or 6 mice in each experimental group in each experiment, and 3 or 4 mice were housed in each cage. When 4 mice were used in each experimental group, two independent experiments were performed and the results from these two experiments were combined. The numbers of mice were determined based on the power analysis. A total of 43 mice were used in the experiments described in this manuscript. Prior to initiating the first experimental procedure in each experiment, mice of the same gender in different cages were placed in one cage and thereafter randomly re-distributed into cages. To perform the experiments in clean and consistent conditions, mice were housed under specific pathogen-free conditions using sterile cages with sterile drinking water and sterile chows. Animal housing rooms were maintained at 22 °C under 14/10 h of light/dark cycle. Mice were monitored once daily. All personnel involved in handling mice and performing experimental procedures had appropriate trainings and technical skills. Every effort was made to minimize distress during experimental procedure. The studies were performed in accordance with approved protocols from the Institutional Animal Care and Use Committee (protocol #2020-3646, approval date of the original version: 19 October 2020, approval date of the most recent version: 10 July 2026).

### 2.2. Infection with T. gondii

*T. gondii* has three major genotypes (types I, II, and III), and genotype II is the most predominant among this parasite isolated from AIDS patients in Europe [22]. The cysts of the ME49 strain (the genotype II) of *T. gondii* were obtained from the brains of Swiss-Webster mice (Taconic Biosciences, Rensselaer, NY) that had been chronically infected after an intraperitoneal injection of 10 cysts, and males and females of the SCID mice were infected with 10 cysts orally (the natural route of infection with this parasite) obtained from the infected Swiss-Webster mice by gavage. Those SCID mice were treated with sulfadiazine (sulfadiazine sodium salt, Sigma-Aldrich, Burlington, MA) (400 mg/L) in drinking water beginning at 8 days after infection for 8 days to control tachyzoite growth during the acute stage of infection and establish a chronic infection in their brains. Thereafter, sulfadiazine was discontinued to initiate reactivation of the chronic cerebral infection with *T. gondii*. The brains of males and females of SCID mice were harvested at 3, 5, or 6 days after discontinuation of sulfadiazine for analyses. One half of each brain was immediately frozen with dry ice for RNA purification, and the other half was fixed in a solution containing 10% formalin, 5% acetic acid, and 70% ethanol for immunohistochemical analyses.

### 2.3. RNA Purification from the Brain and Real-Time RT-PCR

RNA was purified from half of the brain of each mouse using RNA STAT-60 (Tel-test, Friendswood, TX, USA) and treated with DNase I (Invitrogen, Waltham, MA, USA) to remove genomic DNA contamination in the purified RNA, as we described previously [23,24]. cDNA was synthesized from 1 μg of the DNase I-treated RNA from each brain sample. Quantitative PCR reactions were performed with the cDNA using a StepOnePlus real-time PCR system (Applied Biosystems, Branchburg, NJ, USA) with Taqman reagents (Applied Biosystems) [24,25]. Primers and probes for mouse β-actin (a house-keeping control molecule), IFN-γ, inducible nitric oxide synthase (NOS2), indoleamine-2, 3-dioxigenase (IDO1), immunity-related GTPase m3 (Irgm3), guanylate binding protein 1 (Gbp1), IFN-γ receptor 1 (IFNGR1), STAT1, IL-1β, TNF-α, and Arg1 were ready-made products from Applied Biosystems. Primers and probe for tachyzoite-specific SAG1 and LDH1 and bradyzoite-specific BAG1 were also from Applied Biosystems, and sequences of the primers and probe for SAG1 and BAG1 were described previously [24,26]. The sequences of the primers and probe for LDH1 were not provided by Applied Biosystems. The company provided only the sequence of the amplican produced by the PCR, which is TCTTGAACGACGAAAAGCGCGTCATCCCGTGCAGTGTGTACTGCAACGGAGAGTACGGCTTGAAGGACATGTTCATTGGTCTCCCGGCCGTCATTGGAGGCGCCGGCATCGAGCGCGTCATCGAGCTCGAGCTGAACGAGGAGGAGAAG. Amounts of mRNA for the target molecules of interest were normalized to amounts of mRNA for β-actin. The total amounts of RNA purified from the brain of each mouse and expression levels of mRNA for β-actin in those RNA preparations were consistent in individual mice and did not differ between the sexes of mice, the timings of obtaining their brains after initiation of reactivation of *T. gondii* infection, and the levels of tachyzoite burden.

### 2.4. Immunohistochemical Staining for T. gondii

The fixed brains of infected SCID mice were embedded in paraffin, and sagittal sections (4 μm thickness) of the paraffin-embedded brains were stained with rabbit polyclonal anti-*T. gondii* antibodies [24,27] using a Ventana Discovery Ultra instrument (Roche Diagnostics, Indianapolis, IN, USA). After deparaffinization and antigen retrieval with Ventana CC1 (Roche), the slides were incubated with anti-*T. gondii* antibody at 1:1000 dilution at 37 °C for 1 h. The slides were then incubated with Ventana anti-rabbit-HQ (Roche) for 20 min, followed by incubation with Ventana anti-HQ-HRP (Roche). The staining was then amplified using Ventana’s Discovery TSA Amplification Kit (Roche) for 16 min, followed by linking with Discovery Amplification Multimer-HRP (Roche) for 20 min and DAB detection. Slides were counterstained with Meyer’s hematoxylin, blued, and mounted.

Number of areas associated with tachyzoite proliferation in sagittal sections of each brain was measured by scanning the entire field of the sagittal section at ×100 and ×200 magnifications. Those sagittal sections display the areas in the middle of each brain and cover the entire field of the brain including cerebrum and cerebellum. Two sections with at least 40 μm distance from each brain were applied for the counting, and the average number of the areas associated with tachyzoite growth from those two sagittal sections was used for each mouse.

### 2.5. Statistical Analysis

Levels of significance in differences between experimental groups were determined by Student’s *t* test or Mann–Whitney *U* test using GraphPad Prism software 11.0. Differences that had *p* values <0.05 were considered significant.

## 3. Results

### 3.1. Female SCID Mice Are More Susceptible than the Males to Reactivation of Cerebral Infection with T. gondii

**Figure 1:**
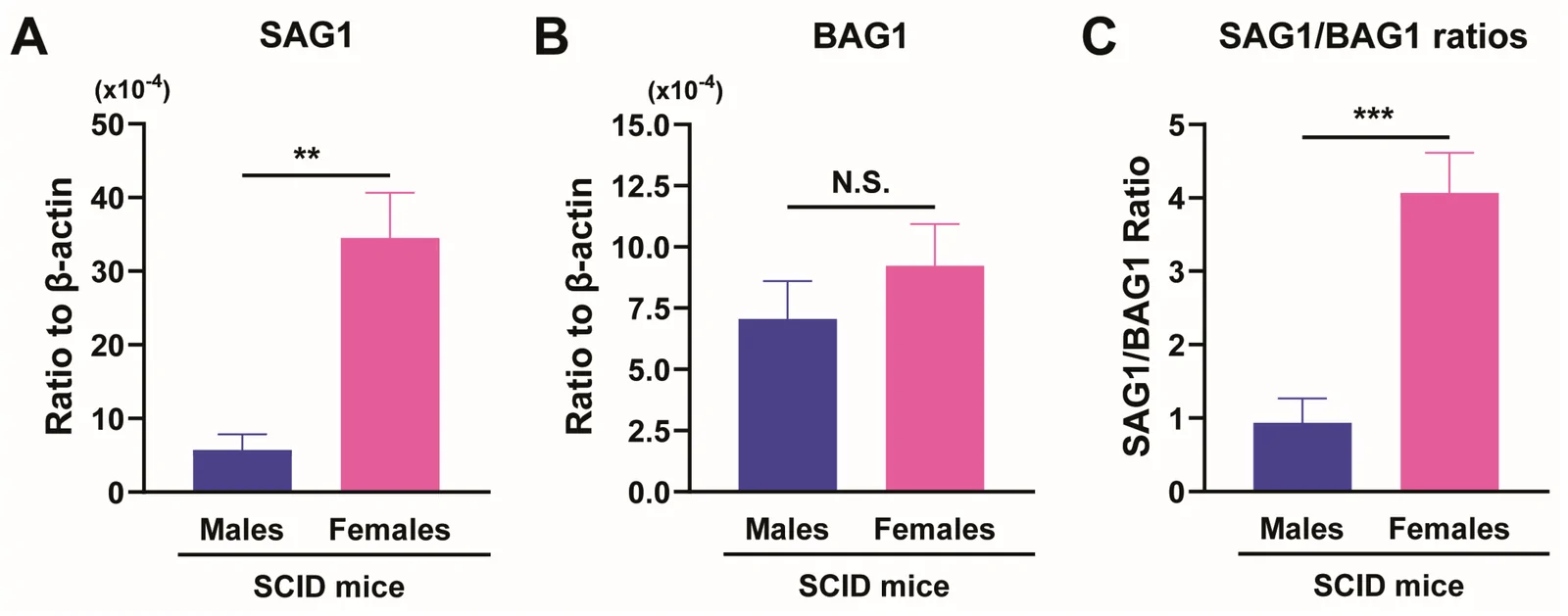
Female SCID mice have greater mRNA levels for tachyzoite-specific SAG1 in their brains than the males during reactivation of cerebral infection with *T. gondii*. Males and females of SCID mice were infected with 10 cysts of *T. gondii* orally by gavage. Mice were treated with sulfadiazine (400 mg/L) in drinking water beginning at 8 days after infection for 8 days to control tachyzoite growth during the acute stage of infection and establish a chronic infection in their brains. Discontinuation of sulfadiazine in these mice initiates reactivation of the infection in their brains. At 6 days after discontinuation of sulfadiazine, mRNA levels for (**A**) tachyzoite-specific SAG1 and (**B**) bradyzoite-specific BAG1 in their brains were measured by real-time RT-PCR. (**C**) The ratios of mRNA levels for tachyzoite-specific SAG to bradyzoite-specific BAG1 mRNA levels were determined to indicate the degree of reactivation of the cerebral *T. gondii* infection. The figure shows the mean ± SE. N = 6 in each gender. ** *p* < 0.01, *** *p*< 0.001. N.S., not significant.

Male and female SCID mice deficient in both T and B cells were infected with *T. gondii* and treated with sulfadiazine beginning at 8 days after infection for 8 days to control tachyzoite proliferation during the acute stage of infection and establish chronic infection in their brains. At 6 days after discontinuation of sulfadiazine, which initiates reactivation of the infection, six times greater levels of mRNA for tachyzoite-specific SAG1 were detected in the brains of the females than males (*p* < 0.01, Figure 1A). In contrast, mRNA levels for bradyzoite (cyst)-specific BAG1 did not statistically differ between the brains of the males and females, although the females had about 30% greater levels of mRNA levels for this molecule than the males (Figure 1B). Reactivation of chronic *T. gondii* infection in the brain is initiated by rupture of cysts, followed by conversion of the bradyzoites released from the ruptured cysts to tachyzoites, and proliferation of the tachyzoites. Therefore, the ratio of tachyzoite-specific SAG1 mRNA levels (in comparison with β-actin mRNA levels) to bradyzoite-specific BAG1 mRNA levels (in comparison with β-actin mRNA levels) can be an indicator for the degree of reactivation of cerebral *T. gondii* infection. Notably, the ratios of SAG1 mRNA levels to BAG mRNA levels were four times greater in the brains of the females than the males (*p* < 0.001, Figure 1C). These results indicate that accelerated conversion from the cysts to tachyzoites and proliferation of the tachyzoites occurred in the brains of female SCID mice when compared to the males during reactivation of *T. gondii* infection.

### 3.2. The Increased Tachyzoite Burden in the Brains of Female SCID Mice Occurs Under the Presence of Cerebral Expression of mRNA for IFN-γ and the Effector Molecules of IFN-γ-Mediated Protective Immunity Against Tachyzoites Equivalent to in the Males During Reactivation of Chronic T. gondii Infection

**Figure 2:**
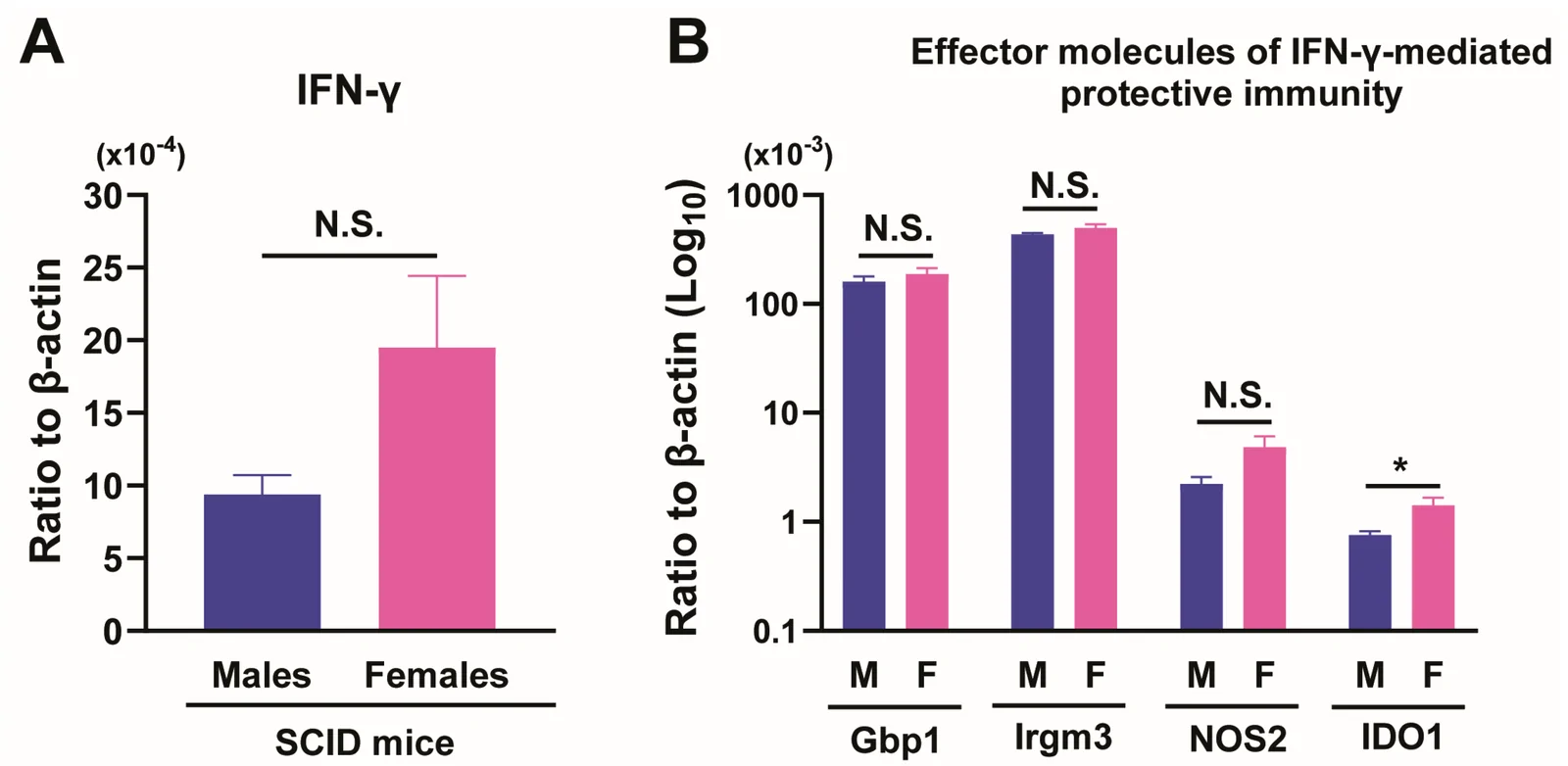
Cerebral mRNA levels for IFN-γ and effector molecules of the IFN-γ-mediated protective immunity against tachyzoites do not differ between males and females of SCID mice during reactivation of *T. gondii* infection. Males and females of SCID mice were infected with 10 cysts of *T. gondii* orally by gavage. Mice were treated with sulfadiazine (400 mg/L) in drinking water beginning at 8 days after infection for 8 days to control tachyzoite growth during the acute stage of infection and establish a chronic infection in their brains. Discontinuation of sulfadiazine in these mice initiates reactivation of the infection in their brains. At 6 days after discontinuation of sulfadiazine, mRNA levels for (**A**) IFN-γ and (**B**) effector molecules of the IFN-γ-mediated protective immunity (Gbp1, Irgm3, NOS2, and IDO1) in their brains were measured by real-time RT-PCR. The “M” and “F” in the panel B mean “males” and “females”, respectively. The figure shows the mean ± SE. N = 6 in each gender. * *p* < 0.05. N.S., not significant.

IFN-γ is the key mediator of the protective immunity to prevent proliferation of tachyzoites in the brain [18,19]. Since greater levels of tachyzoite-specific SAG1 mRNA were detected in the brains of females than males of SCID mice during reactivation of *T. gondii* infection, we examined mRNA levels for IFN-γ in the brains of these mice during reactivation of the infection. At 6 days after initiation of reactivation of the infection, cerebral IFN-γ mRNA levels were approximately twice as high in the females than males, although the difference did not reach statistical significance due to variations in the levels among individual animals especially in the females (*p* = 0.076, Figure 2A).

The protective effects of IFN-γ are mediated by activation of expression of the effector molecules capable of inhibiting tachyzoite growth. Those effector molecules include IFN-γ-inducible GTPases such as Irgm3 and Gbp1 that accumulate to and disrupt the parasitophorous vacuole, in which tachyzoites reside and proliferate in infected host cells, to kill of the tachyzoites [28,29,30], NOS2 that produces nitric oxide to kill intracellular tachyzoites [31], and IDO1 that catalyzes L-tryptophan required for tachyzoite proliferation [32]. Cerebral mRNA levels for Gbp1, Irgm3, and NOS2 were equivalent between the males and females of the SCID mice and did not differ between the sexes of these mice (Figure 2B). The mRNA levels for IDO1 in the females were slightly higher than those of the males, and this difference reached statistical significance (*p* < 0.05, Figure 2B).

**Figure 3:**
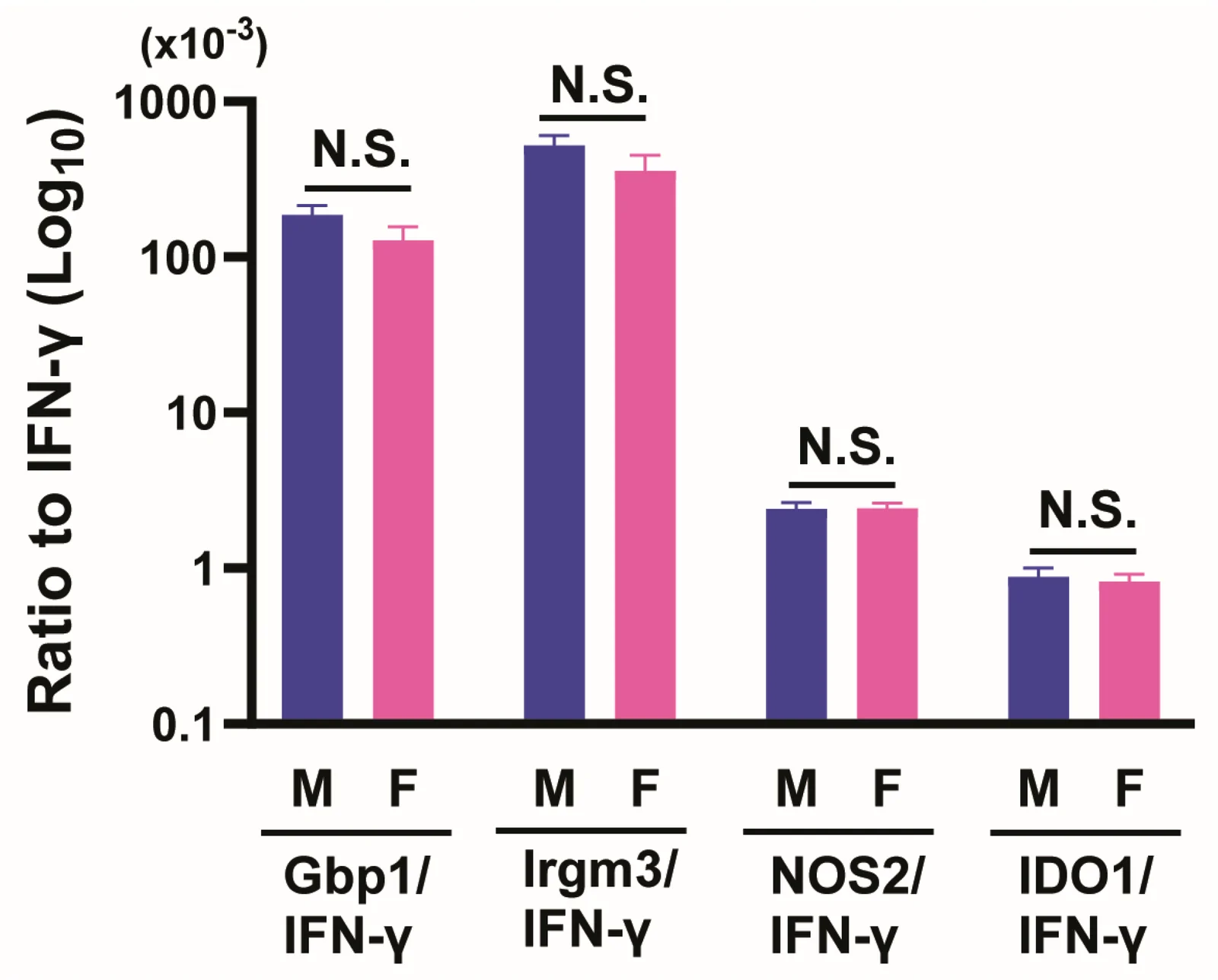
The ratios of mRNA for effector molecules (Gbp1, Irgm3, NOS2, and IDO1) of the IFN-γ-mediated protective immunity against tachyzoites to mRNA levels for IFN-γ do not differ between the brains of males and females of SCID mice during reactivation of cerebral *T. gondii* infection. Males and females of SCID mice were infected with 10 cysts of *T. gondii* orally by gavage. Mice were treated with sulfadiazine (400 mg/L) in drinking water beginning at 8 days after infection for 8 days to control tachyzoite growth during the acute stage of infection and establish a chronic infection in their brains. Discontinuation of sulfadiazine in these mice initiates reactivation of the infection in their brains. At 6 days after discontinuation of sulfadiazine, the ratios of mRNA levels for each of Gbp1, Irgm3, NOS2, and IDO1 to mRNA levels for IFN-γ were measured by real-time RT-PCR. The “M” and “F” in the Figure mean “males” and “females”, respectively. The figure shows the mean ± SE. N = 6 in each gender. N.S., not significant.

To address the efficiency of activating the expressions of those effector molecules by IFN-γ in the brains of males and females of SCID mice during reactivation of cerebral *T. gondii* infection, we also compared the ratios of mRNA levels for the each of the effector molecules to IFN-γ mRNA levels in the brains of these mice. Those ratios did not differ between males and females of the SCID mice during reactivation of *T. gondii* infection (Figure 3). These results suggest that the accelerated tachyzoite proliferation occurs in the brains of female SCID mice when compared to the males under the presence of equivalently activated IFN-γ-mediated protective immunity in the males and the females.

### 3.3. Efficiency of IFN-γ-Mediated Protective Immunity to Inhibit Tachyzoite Proliferation Is Lower in the Brains of Females than Males of SCID Mice

**Figure 4:**
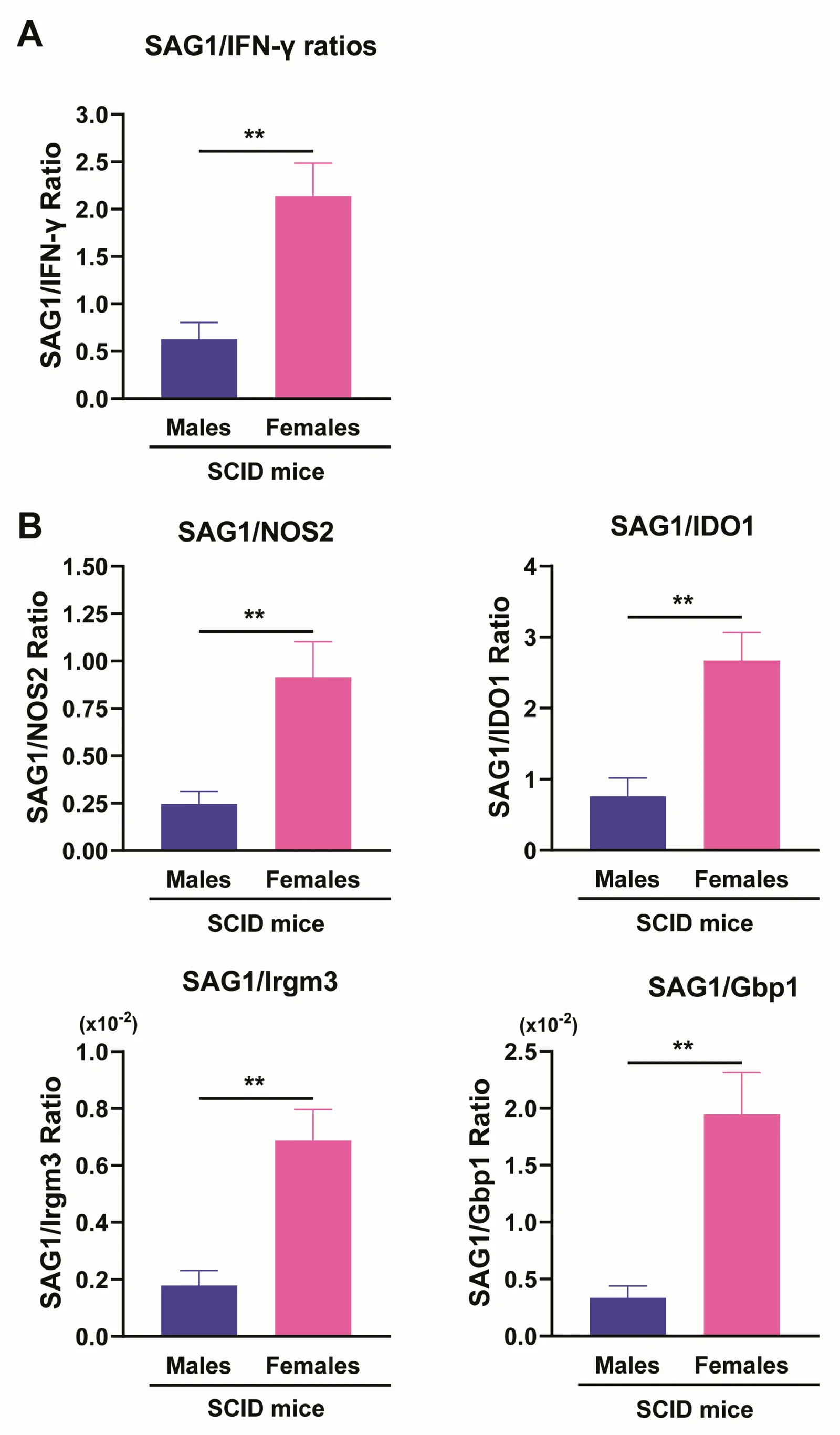
The ratios of tachyzoite-specific SAG1 mRNA levels to mRNA levels for IFN-γ and each of the four effector molecules (NOS2, IDO1, Irgm3, and Gbp1) of the IFN-γ-mediated protective immunity against tachyzoites are greater in the brains of females than males of SCID mice during reactivation of chronic *T. gondii* infection. Males and females of SCID mice were infected with 10 cysts of *T. gondii* orally by gavage. Mice were treated with sulfadiazine (400 mg/L) in drinking water beginning at 8 days after infection for 8 days to control tachyzoite growth during the acute stage of infection and establish a chronic infection in their brains. At 6 days after initiation of reactivation of the infection by discontinuation of sulfadiazine, the mRNA levels for SAG1, IFN-γ, NOS2, IDO1, Irgm3, and Gbp1 in their brains were measured by real-time RT-PCR, and the ratios of tachyzoite-specific SAG1 mRNA levels to mRNA levels for each of IFN-γ, NOS2, IDO1, Irgm3, and Gbp1 were calculated to indicate the efficiency of the mediators of the protective immunity to restrict cerebral tachyzoite proliferation. (**A**) The ratios of SAG1 mRNA levels to IFN-γ mRNA levels. (**B**) The ratios of SAG1 mRNA levels to mRNA levels for each of NOS2, IDO1, Irgm3 and Gbp1. The figure shows the mean ± SE. N = 6 in each of each gender. ** *p* < 0.01.

To address the efficiency of IFN-γ-mediated protective innate immunity to control tachyzoite proliferation in the brains of males and females of the SCID mice during reactivation of *T. gondii* infection, we compared the ratios of SAG1 mRNA levels (in comparison with β-actin mRNA levels) to IFN-γ mRNA levels (in comparison with β-actin mRNA levels) in their brains. Notably, the ratios of SAG1 mRNA levels to IFN-γ mRNA levels were three times greater in the brains of female SCID mice than those of the males (*p* < 0.01, Figure 4A). We also measured the ratios of SAG1 mRNA levels to mRNA levels for each of the effector molecules in the anti-tachyzoite IFN-γ-mediated protective immunity (NOS2, IDO1, Irgm3, and Gbp1) in their brains at 6 days after initiation of reactivation of the infection. Those ratios were approximately 3.5 times to 6 times greater in the brains of female SCID mice than those of the males (Figure 4B) (*p* < 0.01 for each of the four effector molecules). These results indicate that the IFN-γ-mediated protective innate immunity in the brain of the female SCID mice is markedly less efficient in suppressing cerebral tachyzoite proliferation than those of males during reactivation of *T. gondii* infection.

### 3.4. Accelerated Tachyzoite Proliferation Occurs in Female SCID Mice When Compared to the Males During the Progression of Reactivation of T. gondii Infection

**Figure 5:**
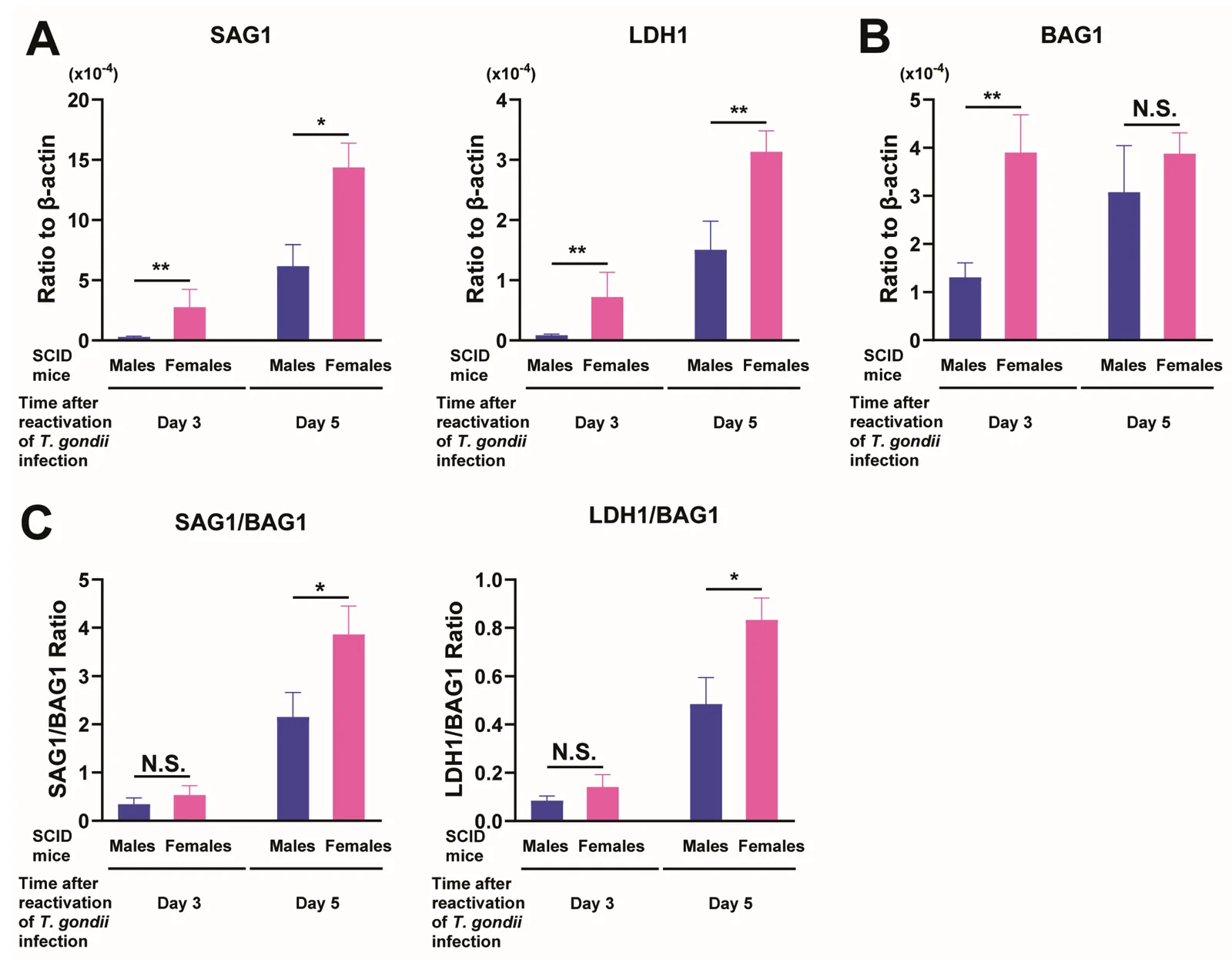
Accelerated cerebral tachyzoite proliferation occurs in females when compared to males of SCID mice especially during the progression of reactivation of *T. gondii* infection. Males and females of SCID mice were infected with 10 cysts of *T. gondii* orally by gavage, and treated with sulfadiazine (400 mg/L) in drinking water beginning at 8 days after infection for 8 days to control tachyzoite growth during the acute stage of infection and establish a chronic infection in their brains. At 3 and 5 days after initiation of reactivation of the infection by discontinuation of sulfadiazine, mRNA levels for tachyzoite-specific SAG1 and LDH1 and bradyzoite-specific BAG1 in their brains were measured by real-time RT-PCR, and the ratios of SAG1 and LDH1 mRNA levels to BAG1 mRNA levels were calculated to indicate the degrees of reactivation of cerebral *T. gondii* infection. (**A**) Cerebral mRNA levels for SAG1 and LDH1. (**B**) Cerebral BAG1 mRNA levels. (**C**) The ratios of SAG1 and LDH1 mRNA levels to BAG1 mRNA levels. The results from two independent experiments were combined. The figure shows the mean ± SE. N = 7 or 8 for each gender. * *p* < 0.05, ** *p* < 0.01. N.S., not significant.

We performed a time-course study to further elucidate the differences in the sequence of cerebral tachyzoite growth between males and females of SCID mice during reactivation of *T. gondii* infection. In this study, we compared mRNA levels for not only SAG1 (a tachyzoite-specific cell surface molecule) but also for LDH1 (a tachyzoite-specific intracellular enzyme) in the brains of males and females of SCID mice at 3 and 5 days after initiation of reactivation of cerebral *T. gondii* infection. At an early stage (day 3) of reactivation of the infection, low levels of mRNA for tachyzoite-specific SAG1 and LDH1 were detected in the brains of both females and males of the SCID mice, but those levels were significantly greater in the females than the males (*p* < 0.01, Figure 5A). At 5 days of reactivation of the infection, much greater levels of cerebral mRNA levels for both SAG1 and LDH1 were detected in both males and females of the SCID mice when compared to day 3 (Figure 5A), and those mRNA levels were approximately twice as high in the females than the males for both SAG1 and LDH1 (*p* < 0.05 for SAG1 and *p* < 0.01 for LDH1, Figure 5A).

In contrast to those tachyzoite-specific molecules, bradyzoite-specific BAG1 mRNA levels did not differ between males and females of SCID mice at day 5 of reactivation of *T. gondii* infection (Figure 5B), which is consistent with those levels at day 6 of reactivation of the infection shown in Figure 1B. However, BAG1 mRNA levels at day 3 were more than two times greater in the brains of the females than the males of the SCID mice (*p* < 0.01, Figure 5B). These gender-dependent differences in the BAG1 mRNA levels between day 3 and day 5 are mostly due to 2.5-fold increases in BAG1 mRNA levels in the males from day 3 to day 5, although this difference did not reach statistical significance (*p* = 0.1026). In contrast, BAG1 mRNA levels in the females remained comparable between these two time points after initiation of reactivation of *T. gondii* infection (Figure 5B). These results may suggest that large portions of tachyzoites proliferating in the brains of male SCID mice converted to cysts (bradyzoites) during the time period between day 3 and day 5 of reactivation of *T. gondii* infection, whereas tachyzoites mostly kept proliferating with few conversions to cysts in the females during this time point.

When the reactivation indexes (the ratios of tachyzoite-specific SAG1 and LDH1 mRNA levels to bradyzoite-specific BAG1 mRNA levels) were compared between the brains of males and females of the SCID mice at day 3 and day 5 of reactivation of *T. gondii* infection, those reactivation indexes were nearly twice as high in both SAG1 and LDH1 in the females than the males at day 5 of reactivation of the infection (*p* < 0.05, Figure 5C), whereas these index values did not differ between the sexes at day 3 of reactivation of the infection (Figure 5C). These results indicate that cerebral tachyzoite proliferation becomes markedly accelerated in female SCID mice when compared to the males during the progression from day 3 to day 5 of reactivation of cerebral *T. gondii* infection.

### 3.5. Greater Numbers of the Areas Associated with Tachyzoite Proliferation Are Present in the Brains of Female SCID Mice than of the Males During Reactivation of T. gondii Infection

**Figure 6:**
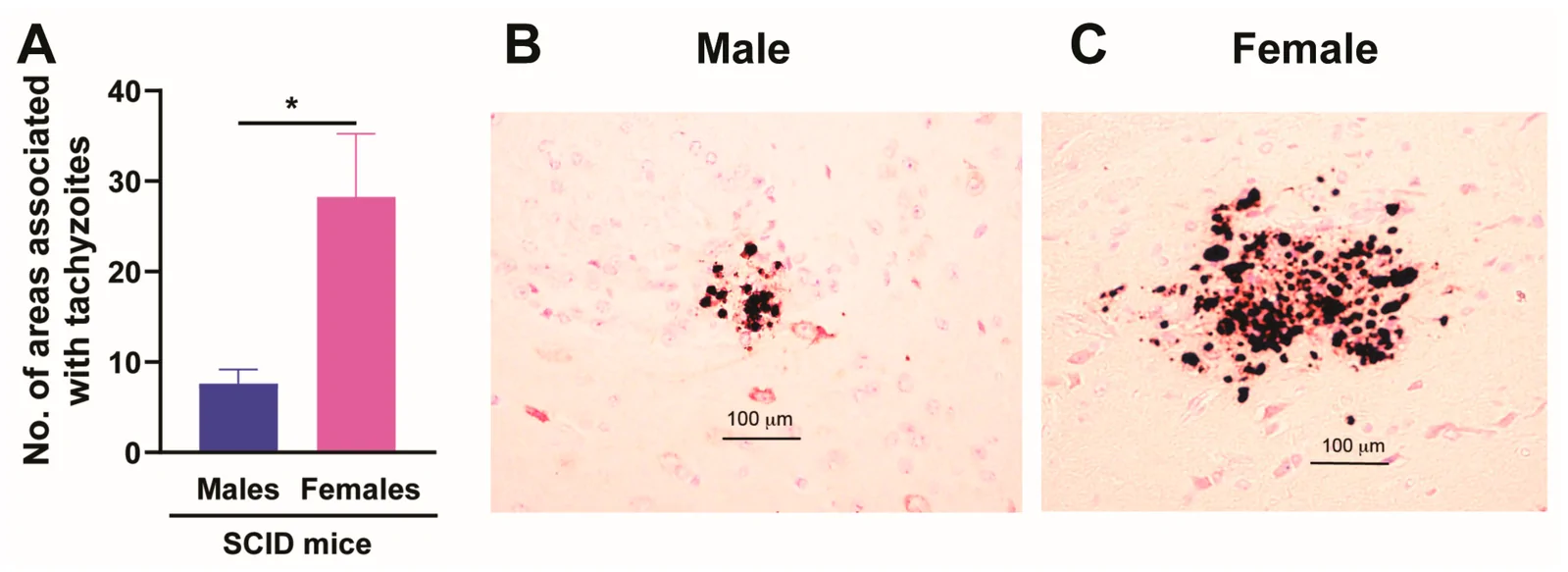
Greater numbers of areas associated with tachyzoite growth are present in the brains of female SCID mice than of the males during reactivation of *T. gondii* infection. Males and females of SCID mice were infected with 10 cysts of *T. gondii* orally by gavage and treated with sulfadiazine (400 mg/L) in drinking water beginning at 8 days after infection for 8 days to control tachyzoite growth during the acute stage of infection and establish a chronic infection in their brains. At 5 days after initiation of reactivation of the infection by discontinuation of sulfadiazine, their brains were fixed, and sagittal sections of the brains were applied to immunohistochemical staining for *T. gondii*. Two sagittal sections with at least 40 μm distance from the brain of each mouse were stained for the parasite, and numbers of areas associated with tachyzoite proliferation in the entire fields of each of the sagittal sections were microscopically counted using both ×100 and ×200 magnifications. The mean value from the counts from two sagittal sections was used for each mouse in the analysis. (**A**) Numbers of areas associated with tachyzoites detected in the sagittal section of the brains of females and males of the SCID mice. A representative image of the area associated with tachyzoites in the immunohistochemically stained, sagittal sections of the brain of (**B**) males and (**C**) females of the SCID mice. Panel A shows the mean ± SE. N = 4 mice in each of females and males of SCID mice. * *p* < 0.05.

We performed immunohistochemical analyses to visualize proliferation of tachyzoites in the brains of males and females of SCID mice at day 5 of reactivation of *T. gondii* infection. More than three times greater numbers of areas associated with tachyzoite proliferation were detected in the parenchyma of the brains of female SCID mice than those of the males (*p* < 0.05, Figure 6A). Furthermore, whereas relatively small numbers of tachyzoites were detected in many of these areas in the brains of the males (a representative of those areas is shown in Figure 6B), a majority of those areas in the brains of the females were associated with large numbers of tachyzoites (a representative of those areas is shown in Figure 6C). Locations of the foci associated with tachyzoite growth within the brain parenchyma did not differ between the sexes of the SCID mice. These results further depict that much more accelerated tachyzoite proliferation occurred in the brains of female SCID mice than in the males during the progression of reactivation of *T. gondii* infection.

### 3.6. Accelerated Cerebral Tachyzoite Growth in Female SCID Mice During the Progression of Reactivation of T. gondii Infection Occurs Despite the Presence of Similarly Elevated IFN-γ mRNA Levels in Both Females and Males

**Figure 7:**
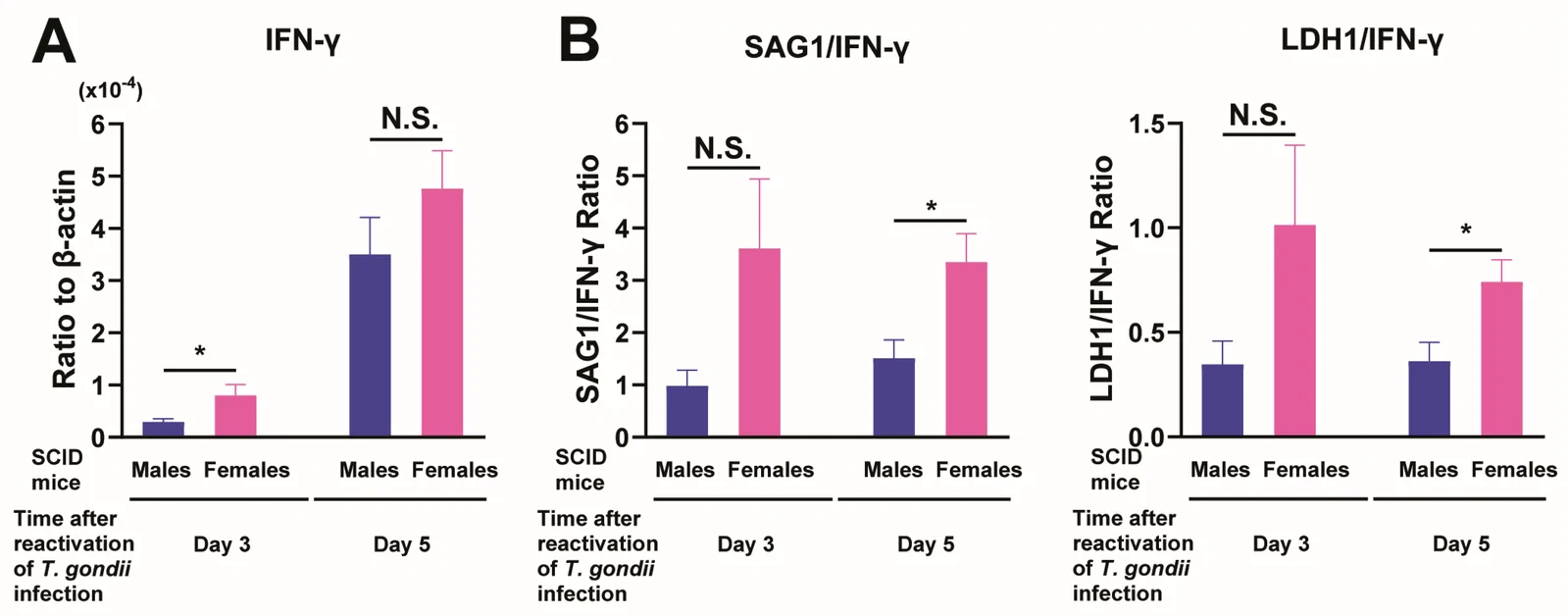
The efficiency of the IFN-γ-mediated protective innate immunity to restrict cerebral tachyzoite growth is lower in females than males of SCID mice during the progression of reactivation of *T. gondii* infection. Males and females of SCID mice were infected with 10 cysts of *T. gondii* orally by gavage and treated with sulfadiazine (400 mg/L) in drinking water beginning at 8 days after infection for 8 days to control tachyzoite growth during the acute stage of infection and establish a chronic infection in their brains. At 3 and 5 days after initiation of reactivation of the infection by discontinuation of sulfadiazine, mRNA levels for IFN-γ and tachyzoite-specific SAG1 and LDH1 in their brains were measured by real-time RT-PCR, and the ratios of mRNA levels for tachyzoite-specific SAG1 and LDH1 to IFN-γ mRNA levels in their brains were calculated. (**A**) Cerebral IFN-γ mRNA levels. (**B**) The ratios of mRNA levels for SAG1 and LDH1 to IFN-γ mRNA levels. The results from two independent experiments were combined. The figure shows the mean ± SE. N = 7 or 8 for each gender. * *p* < 0.05. N.S., not significant.

We compared IFN-γ mRNA levels in the brains of females and males of SCID mice at day 3 and day 5 after initiation of reactivation of *T. gondii* infection. At day 3, IFN-γ mRNA levels were relatively low in the brains of both the males and the females but those levels were significantly higher in the females than the males (*p* < 0.05, Figure 7A). At day 5, significantly more increases in cerebral IFN-γ mRNA were detected in both males and females of the SCID mice when compared to day 3 (Figure 7A), and these levels did not differ between the males and the females.

When the ratios of mRNA levels for tachyzoite-specific SAG1 and LDH1 to IFN-γ mRNA levels were compared between females and males of the SCID mice, these ratios were approximately two times greater in the brains of the females than the males for each of SAG1 and LDH1 at day 5 after initiation of reactivation of *T. gondii* infection (*p* < 0.05) (Figure 7B). These results are consistent with those observed at day 6 after initiation of reactivation of the infection shown in Figure 4A. At day 3, the ratios of SAG1 and LDH1 mRNA levels to IFN-γ mRNA levels also tended to be greater in the females than the males, and these ratios did not reach statistical significance mostly due to large variations in the values among individuals in the females (Figure 7B). These results indicate that despite the presence of equivalently increased levels of mRNA for IFN-γ in the brains of both males and females of infected SCID mice, the IFN-γ-mediated protective innate immunity in the brains of the females was less effective in restricting tachyzoite growth than that in the males during the progression (from day 3 to day 5) of reactivation of cerebral *T. gondii* infection.

### 3.7. Expression Levels of mRNA for IFN-γ Receptor and STAT1, the Key IFN-γ-Mediated Signaling Molecule, Are Comparable in the Brains of Males and Females of SCID Mice During Reactivation of T. gondii Infection

**Figure 8:**
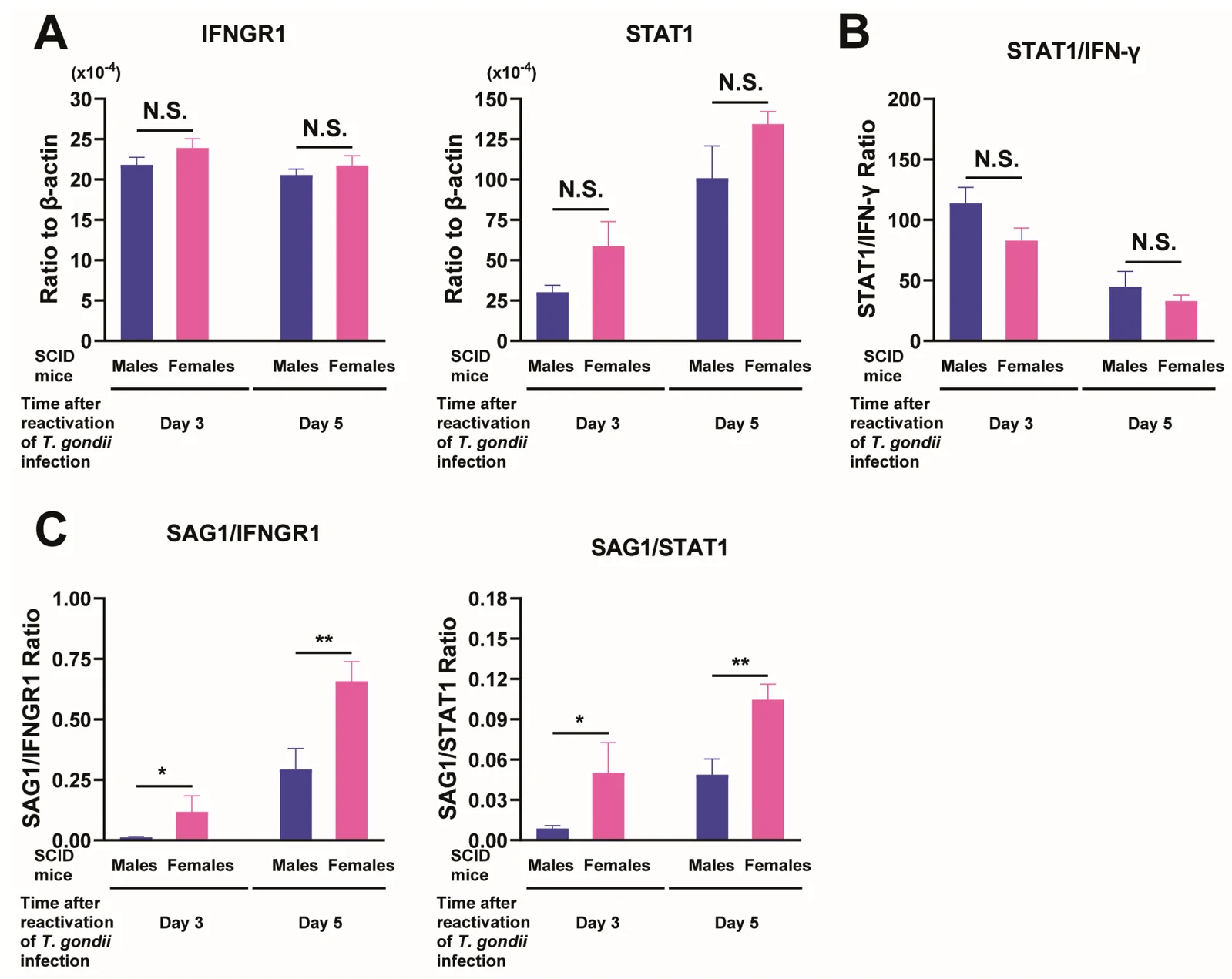
Expression levels of mRNA for IFNGR1 and STAT1, the key IFN-γ-mediated signaling molecule, do not differ between the brains of males and females of SCID mice but the efficiency of those molecules to inhibit tachyzoite growth is lower in the females than the males during reactivation of *T. gondii* infection. Males and females of SCID mice were infected with 10 cysts of *T. gondii* orally by gavage and treated with sulfadiazine (400 mg/L) in drinking water beginning at 8 days after infection for 8 days to control tachyzoite growth during the acute stage of infection and establish a chronic infection in their brains. Discontinuation of sulfadiazine in these mice initiates reactivation of the infection in their brains. At 3 and 5 days after discontinuation of sulfadiazine, mRNA levels for IFNGR1 and STAT1 were measured by real-time RT-PCR. The ratios of STAT1 mRNA levels to IFN-γ mRNA levels and the ratios of tachyzoite-specific SAG1 mRNA levels to mRNA levels for IFNG1 and STAT1 were also calculated. (**A**) mRNA levels for IFNGR1 and STAT1. (**B**) The ratios of STAT1 mRNA levels to IFN-γ mRNA levels. (**C**) The ratios of tachyzoite-specific SAG1 mRNA levels to mRNA levels for IFNGR1 and STAT1. The figure shows the mean ± SE. N = 7 or 8 in each gender. * *p* < 0.05 and ** *p* < 0.01. N.S., not significant.

IFN-γ activates the effector innate immune cells by binding to the IFN-γ receptor expressed on the surface of those effector cells, and its binding to the receptor activates the signaling pathway to activate their protective functions to inhibit intracellular proliferation of tachyzoites. STAT1 is the key mediator of the IFN-γ signaling pathway [33,34]. Since the IFN-γ-mediated protective immunity became less efficient in inhibiting tachyzoite proliferation in the brains of female SCID mice during the progression stage of reactivation of *T. gondii* infection, we compared expression levels of mRNA for IFNGR1 and STAT1 in the brains of males and females of SCID mice at day 3 and day 5 after initiation of reactivation of the infection. Cerebral mRNA levels for both IFNGR1 and STAT1 did not differ between the genders at both day 3 and day 5 of reactivation of the infection (Figure 8A). Consistently, the ratios of STAT1 mRNA levels to IFN-γ mRNA levels also did not differ between the males and females of the SCID mice at both day 3 and day 5 of reactivation of the infection, although these ratios tended to be slightly higher in the males than the females (Figure 8B). These results suggest that the activation pathway of the IFN-γ-mediated protective immunity was comparably functional in the brains of both males and females of SCID mice during reactivation of *T. gondii* infection. These results are consistent with the presence of equivalent ratios of mRNA levels for the IFN-γ-mediated effector molecules (NOS2, IDO1, Irgm3, and Gbp1) to IFN-γ mRNA levels in males and females of these mice at day 6 of reactivation of *T. gondii* infection shown in Figure 3.

In contrast, when the ratios of SAG1 mRNA levels to each of IFNGR1 and STAT1 mRNA levels were compared between the brains of males and females of SCID mice during the course of reactivation of *T. gondii* infection, these ratios were significantly higher in females than males of the SCID mice at both day 3 and day 5 of reactivation of the infection (*p* < 0.05 at day 3 and *p* < 0.01 at day 5, Figure 8C). Thus, tachyzoites most likely proliferate faster in the brains of females than males of SCID mice despite the presence of an equivalently activated IFN-γ-mediated signaling pathway during reactivation of *T. gondii* infection.

### 3.8. Cerebral TNF-α and IL-1β mRNA Levels Do Not Differ in the Brains of Males and Females of SCID Mice During the Progression of Tachyzoite Growth During Reactivation of T. gondii Infection

**Figure 9:**
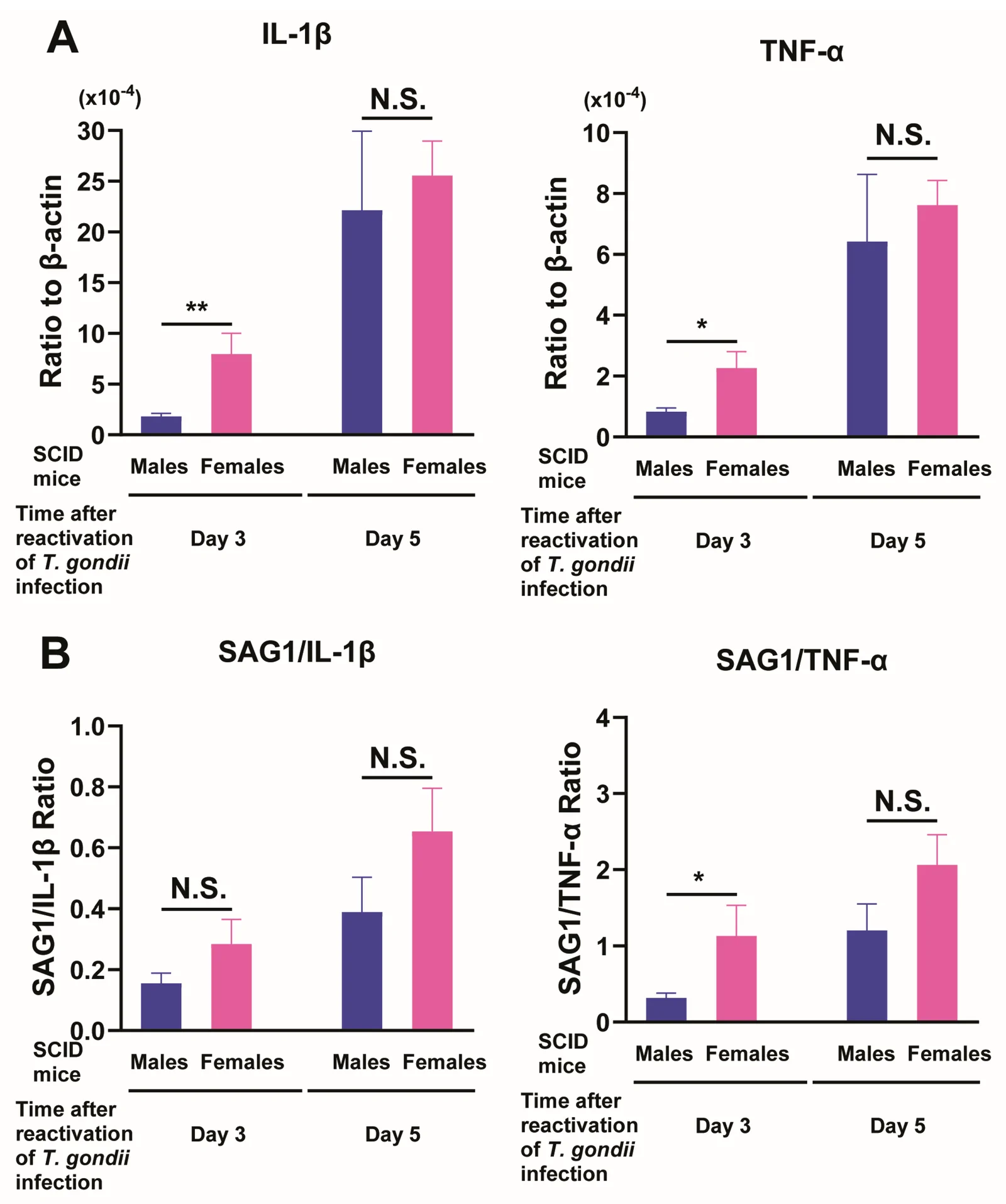
Equivalent IL-1β and TNF-α mRNA levels are present in the brains of males and females of SCID mice during the progression of reactivation of *T. gondii* infection. Males and females of SCID mice were infected with 10 cysts of *T. gondii* orally by gavage and treated with sulfadiazine (400 mg/L) in drinking water beginning at 8 days after infection for 8 days to control tachyzoite growth during the acute stage of infection and establish a chronic infection in their brains. Discontinuation of sulfadiazine in these mice initiates reactivation of the infection in their brains. At 3 and 5 days after discontinuation of sulfadiazine, mRNA levels for IL-1β and TNF-α were measured by real-time RT-PCR. The ratios of tachyzoite-specific SAG1 mRNA levels to mRNA levels for IL-1β and TNF-α were also calculated. (**A**) mRNA levels for IL-1β and TNF-α. (**B**) The ratios of tachyzoite-specific SAG1 mRNA levels to mRNA levels for IL-1β and TNF-α. The figure shows the mean ± SE. N = 7 or 8 in each gender. * *p*< 0.05, ** *p* < 0.01. N.S., not significant.

Whereas IFN-γ plays the key roles in the protective immunity against cerebral *T. gondii* infection [18,19], TNF-α and IL-1β participate in the protection against tachyzoites during *T. gondii* infection [35,36,37]. Therefore, we compared mRNA expression for TNF-α and IL-1β in the brains of males and females of SCID mice during reactivation of *T. gondii* infection. Cerebral expression of mRNA for both IL-1β and TNF-α remained relatively low at day 3 of reactivation of the infection, but those levels were significantly higher in the females than the males (*p* < 0.01 for IL-1β and *p* < 0.05 for TNF-α, Figure 9A). In contrast, at day 5, mRNA levels for these cytokines became much higher in both males and females of the SICID mice, and those levels did not differ between the sexes in these mice (Figure 9A). In addition, the ratios of tachyzoite-specific SAG1 mRNA levels to each of IL-1β and TNF-α mRNA levels in the brains did not differ between the sexes in the SCID mice at day 5 after initiation of reactivation of *T. gondii* infection (Figure 9B), although only the ratios of SAG1mRNA to TNF-α mRNA levels reached statistical significance at day 3 of reactivation of the infection (*p* < 0.05, Figure 9B). These results suggest that IL-1β and TNF-α are not major contributors to the increased tachyzoite proliferation in female SCID mice when compared to the males during the progression of reactivation of *T. gondii* infection.

### 3.9. Greater Levels of Arg1 mRNA in the Brains of Female SCID Mice than the Males Correlate with Enhanced Cerebral Tachyzoite Proliferation of the Females During Reactivation of T. gondii Infection

**Figure 10:**
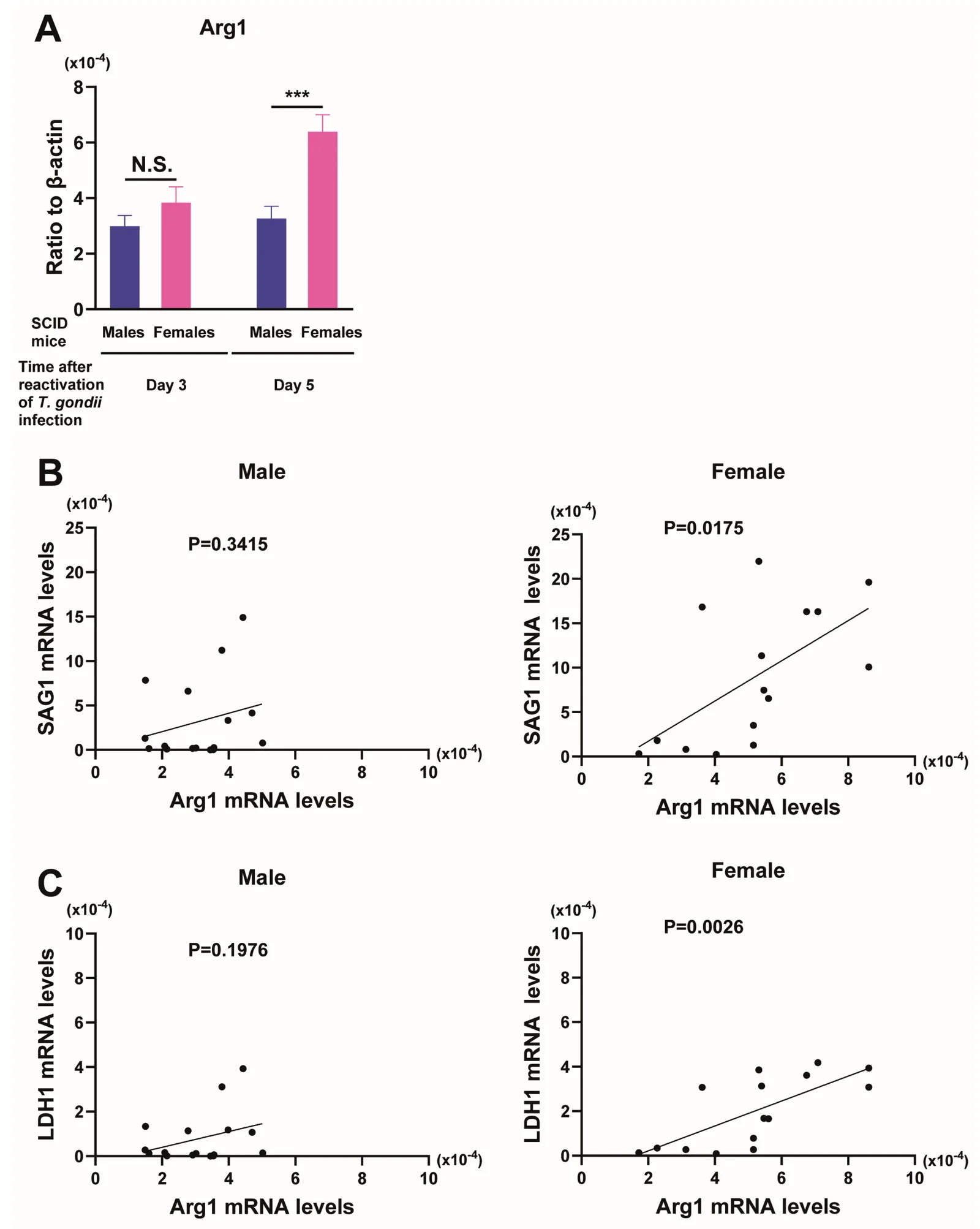
Arg1 mRNA levels are higher in the brains of female SCID mice than the males during the progression of reactivation of *T. gondii* infection, and those increased Arg1 mRNA levels correlate with enhanced cerebral tachyzoite proliferation of only the females during reactivation of the infection. Males and females of SCID mice were infected with 10 cysts of *T. gondii* orally by gavage. Mice were treated with sulfadiazine (400 mg/L) in drinking water beginning at 8 days after infection for 8 days to control tachyzoite growth during the acute stage of infection and establish a chronic infection in their brains. Discontinuation of sulfadiazine in these mice initiates reactivation of the infection in their brains. At 3 and 5 days after discontinuation of sulfadiazine, mRNA levels for Arg1 were measured by real-time RT-PCR. (**A**) mRNA levels for Arg1, (**B**) correlation of Arg1 mRNA levels with tachyzoite-specific SAG1mRNA levels, and (**C**) correlation of Arg1 mRNA levels with tachyzoite-specific LDH1 in the brains of females and males of SCID mice during reactivation of *T. gondii* infection. The panel A shows the mean ± SE. N = 7 or 8 in each gender. *** *p*< 0.001. N.S., not significant.

Arg1 can enhance tachyzoite growth through two different pathways. One is to suppress the protective activity of NOS2 by competing for the availability of L-arginine for their substrate [38,39]. Another is to produce L-ornithine, which is a precursor of a variety of polyamines [40,41,42] that promote tachyzoite growth [43]. Therefore, we compared cerebral mRNA levels for Arg1 between males and females of SCID mice during reactivation of *T. gondii* infection. Significantly greater levels of cerebral Arg1 mRNA were detected in the females than the males at day 5 after initiation of reactivation of the infection (*p* < 0.001, Figure 10A), whereas these levels did not differ between the sexes at day 3 of reactivation of the infection (Figure 10A). The presence of greater expression levels of Arg1 in the females than the males of SCID mice only at day 5 is consistent with the presence of the greater levels of the reactivation index (the ratios of tachyzoite-specific SAG1 and LDH1 mRNA levels to bradyzoite-specific BAG1 mRNA levels) in the former than the latter only at day 5 of reactivation of the infection shown in Figure 5C.

Notably, the increased Arg1 mRNA levels significantly correlated with increased levels of each of tachyzoite-specific SAG1 and LDH1 mRNA levels in the brains of only the females of these mice during reactivation of *T. gondii* infection (*p* = 0.0176 for SAG1, Figure 10B and *p* = 0.0026 for LDH1, Figure 10C). These results strongly suggest that the increased levels of Arg1 in the brains of female SCID mice contribute to the accelerated tachyzoite proliferation in their brains when compared to the males during the progression of reactivation of *T. gondii* infection.

## 4. Discussion

The present study revealed that females of SCID mice deficient in both T and B cells are markedly more susceptible than males of these mice to reactivation of cerebral infection with *T. gondii*. Treatment of infected SCID mice with sulfadiazine during the acute acquired stage of infection can establish a chronic infection with this parasite in their brain, and discontinuation of sulfadiazine initiates reactivation of the cerebral infection. During multiple time points (days 3, 5, and 6) after initiation of reactivation of the infection, female SCID mice had much greater levels of mRNA for tachyzoite-specific SAG1 and/or LDH1 in their brains than the males. Consistently, immunohistochemical analyses revealed the presence of three times greater numbers of the areas associated with tachyzoite growth in the brain parenchyma of female SCID mice than the males at 5 days after the initiation of reactivation of the infection. These results indicate that innate immunity in female SCID mice is less effective than that of the males to restrict cerebral tachyzoite proliferation during reactivation of *T. gondii* infection. To our knowledge, the difference in the efficiency of innate immunity to restrict reactivation of cerebral *T. gondii* infection between genders has not been reported before.

Reactivation of *T. gondii* infection is initiated by rupture of the cysts, which induces release of bradyzoites from those cysts, conversion of the released bradyzoites to tachyzoites, and proliferation of the tachyzoites. Thus, the ratios of tachyzoite-specific SAG1 and LDH1 mRNA levels to bradyzoite-specific BAG1 mRNA levels can indicate the degree of reactivation of *T. gondii* infection in the organ. Notably, the present study uncovered that whereas the ratios of tachyzoite-specific SAG1 and LDH1 mRNA levels to bradyzoite-specific BAG1 mRNA levels did not differ in the brains of females and males of SCID mice at day 3 after initiation of reactivation of *T. gondii* infection, those ratios became nearly twice as high in the brains of the females than the males of those mice at day 5 of reactivation of the infection. Consistently, four-times-greater ratios of SAG1 mRNA levels to BAG1 mRNA levels were detected at day 6 of reactivation of the infection. Thus, a significant acceleration of cerebral tachyzoite proliferation occurs only in female SCID mice during the progression (from day 3 to days 5 and 6) of reactivation of *T. gondii* infection.

The present study also identified that whereas bradyzoite-specific BAG1 mRNA levels were more than twice as high in the brains of females than males of SCID mice at day 3 after initiation of reactivation of *T. gondii* infection, the cerebral BAG1 mRNA levels became comparable between the males and females at day 5 of reactivation of the infection. This difference between day 3 and day 5 was mostly due to 2.5-fold increases in BAG1 mRNA levels from day 3 to day 5 only in the brains of the male mice. Thus, it is most likely that large portions of tachyzoites converted to cysts during the time period from day 3 to day 5 in the brains of male SCID mice, whereas tachyzoites kept proliferating during this time period in the brain of female SCID mice. The continuous proliferation of tachyzoites without converting into cysts would be a major cause of the presence of much greater reactivation ratios (SAG1 and LDH1 mRNA levels to BAG1 mRNA levels) in the brains of the females compared to the males during the progression stage of reactivation of *T. gondii* infection.

Since the present study identified the presence of greater levels of bradyzoite-specific BAG1 mRNA levels in the brains of female SCID mice than the males at the early stage (day 3) of reactivation of *T. gondii* infection, it is most likely that the female mice have more cysts in their brains than the males at the time that reactivation of the infection was initiated (day 0). Therefore, it is possible that the presence of higher levels of mRNA for tachyzoite-specific SAG1 and LDH1 in the females than males of these mice at day 3 is mostly due to the presence of larger numbers of cysts in the former than the latter at the time of initiation of reactivation of *T. gondii* infection. Such greater tachyzoite burden at relatively low levels in the females at the early stage of reactivation of the infection most likely further amplified the accelerated proliferation of the tachyzoites during the progression stage of reactivation of the infection.

A previous study by others using acute acquired infection with *T. gondii* in SCID mice of the *H-2^d^* haplotype demonstrated that the males survived longer than the females although the both died within 3 weeks after infection [44]. They also showed the presence of greater numbers of large necrotic lesions associated with tachyzoite growth in the brains of the females than the males at 15 days after infection [44]. However, since serum levels of IFN-γ were more than 10 times lower in the females than the males during the earlier stage (day 8) of the infection, it is likely that greater levels of tachyzoite growth had occurred in the peripheral organs in the females than the males during the early stage of infection due to the much lower serum levels of IFN-γ in the former, and that the increased tachyzoite proliferation in the periphery in the females had led to migration of greater numbers of tachyzoites to their brain and the presence of larger numbers of the areas associated with tachyzoites in this organ in the females. Consistently, in the present study in which SCID mice were treated with sulfadiazine from day 8 of *T. gondii* infection to inhibit tachyzoite proliferation and establish a chronic infection in their brains, significantly greater levels of bradyzoite (cyst)-specific BAG1 mRNA were detected in the brains of females than males of these mice at the initial stage (day 3) of reactivation of the infection. Those higher levels of BAG1 mRNA most likely reflect migration of larger numbers of tachyzoites into the brains of female SCID mice than the males before the initiation of sulfadiazine treatment 8 days after infection.

IFN-γ is required for prevention of cerebral tachyzoite growth and reactivation of *T. gondii* infection [18,19]. Of interest, despite the occurrence of accelerated cerebral tachyzoite proliferation in females of the SCID mice when compared to the males during reactivation of *T. gondii* infection, IFN-γ mRNA levels in the brain did not differ between sexes of those mice during the progression stage (day 5 and day 6) of reactivation of *T. gondii* infection. When IFN-γ is secreted from its producer cells, this cytokine binds to IFN-γ receptor expressed on the surface of effector cell populations and activates those cells through the signaling pathway initiated by binding of this cytokine to the receptor. STAT1 is the key molecule in the IFN-γ-mediated signaling pathway, and activation of this signaling pathway induces expressions of effector molecules including NOS2, IDO1, Irgm3, and Gbp1 [28,29,30,31,32] as mentioned earlier. Despite an occurrence of the accelerated tachyzoite proliferation in the brains of female SCID mice when compared to the males during reactivation of *T. gondii* infection, cerebral expression levels of mRNA for each of IFN-γ, IFNGR1, STAT1, and the effector molecules did not differ between the sexes of these mice during the progression of reactivation of the infection. In addition, the ratios of mRNA levels for those effector molecules to IFN-γ mRNA levels were comparable in males and females of the SCID mice. These results indicate that IFN-γ-mediated protective innate immunity was comparably activated in both sexes of SCID mice during the progression of reactivation of *T. gondii* infection.

Notably, the ratios of mRNA levels for the tachyzoite-specific SAG1 and/or LDH1 to each of IFN-γ, IFNGR1AT1, and the effector molecules were significantly higher in females than males of SCID mice during the progression stage of reactivation of *T. gondii* infection. These results suggest that tachyzoite proliferation is accelerated in the brains of the female SCID mice when compared to the males during the progression stage of reactivation of the infection under the presence of comparably activated IFN-γ-mediated protective immunity against the parasite. In other words, these results suggest the presence of a pathway that facilitates tachyzoite growth under the presence of the IFN-γ-mediated protective immunity in the brains of female SCID mice during the time when cerebral tachyzoite growth becomes accelerated during reactivation of *T. gondii* infection.

The present study revealed that cerebral expression of Arg1 mRNA becomes significantly greater in females than males of SCID mice at the progression stage (day 5) of reactivation of *T. gondii* infection. Arg1 can facilitate tachyzoite proliferation not only by suppressing the protective activity of NOS2 [38,39] but also producing nutrients that assist proliferation of tachyzoites [43]. Importantly, we identified that increased levels of Arg1 mRNA in the female SCID mice significantly correlate with increased levels of both tachyzoite-specific SAG1 and LDH1 mRNA in their brains during reactivation of *T. gondii* infection. Notably, this correlation between Arg1 mRNA levels and tachyzoite-specific SAG1 and LDH1 mRNA levels did not occur in the brains of the male SCID mice, in which cerebral Arg1 mRNA levels remained significantly lower when compared to the females. Therefore, the increased expression of Arg1 in the brain most likely contributed to the accelerated cerebral tachyzoite growth in the female SCID mice during reactivation of *T. gondii* infection. To our knowledge, a contribution of cerebral Arg1 expression to increasing the susceptibility to reactivation of *T. gondii* infection has not been reported before. The results of the present study do not exclude the possibility of contributions of the immunosuppressive pathways mediated by molecules other than Arg1. It could be possible that Arg1 is one of the pathways that increase the susceptibility of females to reactivation of cerebral *T. gondii* infection.

In addition to *T. gondii* infection, IFN-γ plays critical protective roles in the resistance against cerebral infections with various bacteria [45,46] and viruses [47,48]. Although differences in the susceptibility to cerebral microbial infectious diseases between males and females have been recognized [49,50], we were unable to find any articles reporting gender-dependent differences in the efficiency of IFN-γ-mediated innate immunity in the brain to control a cerebral microbial infection in general. Therefore, the present study provides a novel and valuable insight on the gender-dependent differences in the protective capability of IFN-γ-mediated cerebral innate immunity against a microbial infection.

Epidemiological studies performed in Europe showed an increased risk of female sex in developing TE in AIDS patients [16,17] as mentioned earlier. However, the mechanisms underlying the increased susceptibility of the females to the development of TE remain unknown. Since innate immunity is critical for host defense capability especially in immunocompromised individuals such as those with AIDS with low CD4^+^ T cell counts, it is possible that the lower efficiency of the cerebral IFN-γ-mediated innate immunity in restricting tachyzoite growth in females than males and a potential pathogenic involvement of Arg1 in the female sex-dependent acceleration of the cerebral tachyzoite growth, which is uncovered by the present study, contribute to the increased susceptibility of female AIDS patients to reactivation of cerebral *T. gondii* infection and the development of TE.

## 5. Conclusions

The present study using SCID mice deficient in both T and B lymphocytes revealed that females of these mice are remarkably more susceptible to reactivation of cerebral infection with *T. gondii* than the males. Much greater tachyzoite burden and greater numbers of areas associated with tachyzoite proliferation were detected in the brains of the females than the males during reactivation of the infection. The cerebral tachyzoite growth in the females was especially accelerated along with the progression of reactivation of the infection, which is supported by the evidence of the presence of markedly increased ratios of tachyzoite-specific SAG1 and LDH1 mRNA levels to bradyzoite (cyst)-specific BAG1 mRNA levels in the brains of female SCID mice when compared to the males during the progression of reactivation of *T. gondii* infection. Whereas the indicators of activation of the IFN-γ-mediated protective immunity and expressions of IL-1β and TNF-α were comparable in the brains of the males and females of SCID mice during the progression of reactivation of *T. gondii* infection, significantly greater ratios of tachyzoite-specific SAG1 and LDH1 mRNA to IFN-γ mRNA levels and greater ratios of mRNA levels for those tachyzoite-specific molecules to mRNA levels for each of the four effector molecules (NOS2, IDO1, Irgm3, and Gbp1) of the IFN-γ-mediate protective immunity against tachyzoites were detected in the brains of females than males of SCID mice during the progression stage of reactivation of the infection. Thus, the cerebral IFN-γ-mediated protective innate immunity is less efficient in female than the males of SCID mice in inhibiting tachyzoite proliferation in the brain during the progression of reactivation of *T. gondii* infection. Furthermore, the present study revealed that cerebral mRNA levels for Arg1 became significantly greater in the females than the males when accelerated tachyzoite proliferation occurs in the former during reactivation of the infection, and that the increased Arg1 mRNA levels directly correlated with increased mRNA levels for tachyzoite-specific SAG1 and LDH1 only in the brains of the female SCID mice. Epidemiological studies reported increased risks of female AIDS patients when compared to the males to the development of TE, which is caused by reactivation of chronic infection with *T. gondii*. Thus, it is possible that the decreased efficiency of IFN-γ-mediated protective innate immunity to restrict cerebral tachyzoite growth and a possible pathogenic contribution of Arg1 to the reduced efficiency of the protective innate immunity occur more frequently in females than males of SCID mice and contribute to the increased susceptibility of the former to the development of TE.

## Data Availability

The data generated from this study are presented in this article. Further inquiries can be directed to the corresponding author.

## Abbreviations

IFN-γ	Interferon-gamma
STAT1	Signal transducer and activator of transcription 1
IL-1β	Interleukin 1 beta
TNF-α	Tumor necrosis factor alpha
Arg1	Arginase 1
TE	Toxoplasmic encephalitis
NOS2	Inducible nitric oxide synthase
IDO1	indoleamine-2, 3-dioxigenase
Irgm3	Immunity-related GTPase m3
Gbp1	Guanylate binding protein 1
IFNGR1	IFN-γ receptor 1
SAG1	Surface antigen 1
BAG1	Bradyzoite antigen 1
LDH1	Lactate dehydrogenase 1
